# Supplementary material for: Enhancing the performance of the aggregated bit vector algorithm in network packet classification using GPU
Source: PeerJ Comput Sci. 2019 Apr 15;5:e185. doi: 10.7717/peerj-cs.185 (PMC7924471; doi:10.7717/peerj-cs.185)
Supplement: Supplemental Information 1 — The ABV parallel GPU kernel code is presented. [file peerj-cs-05-185-s001.docx]

#include "stdafx.h"

#include <stdio.h>

#include "Timer.h"

#include <fstream>

#include <iostream>

#include <queue>

#include<stack>

#include <cstring>

#include <string>

#include <bitset>

using namespace std;

typedef struct {

int width;

int height;

unsigned int *elements;

} Matrix;

typedef struct {

int width;

int height;

bool *elements;

} Matrix3;

typedef struct {

int width;

int height;

int *elements;

} Matrix2;

struct Tree {

int data;

int rule;

Tree *left;

Tree *right;

};

struct Tree *newTreeNode(int data)

{

Tree *node = new Tree;

node->data = data;

node->left = NULL;

node->right = NULL;

node->rule = -1;

return node;

}

__global__ void kernel(Tree * Gtree1, Tree * Gtree2, int *out,Matrix headers,Matrix rules,Matrix3 bitvs,Matrix3 bitvd,Matrix3 abitvs,Matrix3 abitvd)

{

for(int i=0;i < ceilf(headers.height/3072)+1;i++){

int index=threadIdx.x + i*3072 + blockIdx.x *768;

if(index<headers.height){

int f0=headers.elements[index+headers.height*0];

int f1=headers.elements[index+headers.height*1];

int f2=headers.elements[index+headers.height*2];

int f3=headers.elements[index+headers.height*3];

int f4=headers.elements[index+headers.height*4];

Tree *tree1;

tree1=Gtree1;

Tree *tree2;

tree2=Gtree2;

Tree *temp;

char src[32];

char dest[32];

int i=0;

for (int j = 32-1; j >= 0; --j) {

if (f0 & (1 << j))

src[i]='1';

else

src[i]='0';

i++;

}

i=0;

for (int j = 32-1; j >= 0; --j) {

if (f1 & (1 << j))

dest[i]='1';

else

dest[i]='0';

i++;

}

//---------------------------------------------------------input Source

char s[1000];

char as[100];

int j=0;

while(j < 32 && tree1 != NULL){

if(tree1->rule != -1)//this node has a bitset

temp=tree1;

if(src[j] == '0' ){

tree1=tree1->left;

} else if(src[j] == '1' ){

tree1=tree1->right;

}

j++;

}

int Ns=temp->rule;

//////////////////////////////////////////////////dst

j=0;

while(j < 32 && tree2 != NULL){

if(tree2->rule != -1)//this node has a bitset

temp=tree2;

if(dest[j] == '0' ){

tree2=tree2->left;

}

else if(dest[j] == '1' ){

tree2=tree2->right;

}

j++;

}

int Nd=temp->rule;

for(int i=0;i<rules.height;i++){

if(bitvs.elements[Ns+bitvs.height*i]==1 && bitvd.elements[Nd+bitvd.height*i]==1)

s[i]='1';

else

s[i]='0';

}

for(int i=0;i<abitvs.width;i++){

if(abitvs.elements[Ns+abitvs.height*i]==1 && abitvd.elements[Nd+abitvd.height*i]==1)

as[i]='1';

else

as[i]='0';

}

j=0;

int f=0;

while(j<abitvs.width && f==0){

if(as[j]=='1'){

for(int i=j*(rules.height/abitvs.width);i<j*(rules.height/abitvs.width)+10;i++){

if(s[i]=='1'){

int nrule=i;

if((f4 == rules.elements[nrule+rules.height*4] || rules.elements[nrule+rules.height*4] == 0)// potocol

&& f2 >= rules.elements[nrule+rules.height*0] //&& sport >= fsport

&& f2 <= rules.elements[nrule+rules.height*1] //&& sport <= esport

&& f3 >= rules.elements[nrule+rules.height*2] //&& dport >= fdport

&& f3 <= rules.elements[nrule+rules.height*3]){ //&& dport <= edport

out[index] = nrule;

f=1;

}

}

}

}//if as

j++;

}//while

}

}//for all headers

}

struct Tree* insertTreeNode(struct Tree *node, int data)

{

if(node == NULL) {

node = newTreeNode(data);

return node;

}

if(data==0 && node->left && node->left->data == 0){

node=node->left;

return node;

}

if(data==1 && node->right && node->right->data == 1){

node=node->right;

return node;

}

if(data == 0 ){

node->left = insertTreeNode(node->left,data);

node=node->left;

}

else if(data == 1 ){

node->right = insertTreeNode(node->right,data);

node=node->right;

}

return node;

}

void traverse(Tree * tree1,Tree * tree2,Tree * p) {

queue<Tree*> q;

if(tree1 == NULL) {

return;

}

q.push(tree1);

int i=-1;

while(!q.empty()) {

Tree *tmpNode = q.front();

q.pop();

i+=1;

tree2[i].data=tmpNode->data;

tree2[i].rule=tmpNode->rule;

if(tmpNode->left!=NULL)

tree2[i].left=(++p);

else

tree2[i].left=NULL;

if(tmpNode->right!=NULL)

tree2[i].right=(++p);

else

tree2[i].right=NULL;

if(tmpNode->left != NULL) {

q.push(tmpNode->left);

}

if(tmpNode->right != NULL) {

q.push(tmpNode->right);

}

}

}

int treeSize(struct Tree *node)

{

if(node == NULL) return 0;

else

return treeSize(node->left) + 1 + treeSize(node->right);

}

string NToB(int str){

string sr;

for (int j = 8-1; j >= 0; --j) {

if (str & (1 << j))

sr+="1";

else

sr+="0";

}

return sr;

}

string IPtoBin(string Ip){

string bin,sub,m;

int x;

bin="";

x=Ip.find('.');

sub=Ip.substr(0,x);

bin+=NToB(atoi(sub.c_str()));

sub=Ip.substr(x+1,Ip.length());

x=x+1+sub.find('.');

sub=sub.substr(0,x);

bin+=NToB(atoi(sub.c_str()));

sub=Ip.substr(x+1,Ip.length());

x=x+1+sub.find('.');

sub=sub.substr(0,x);

bin+=NToB(atoi(sub.c_str()));

sub=Ip.substr(x+1,Ip.length());

x=x+1+sub.find('/');

sub=sub.substr(0,x);

bin+=NToB(atoi(sub.c_str()));

x=Ip.find('/');

sub=Ip.substr(x+1,Ip.length());

Ip=bin.substr(0,atoi(sub.c_str()))+"*";

return Ip;

}

unsigned int BintoInt(string Ip){

if(Ip=="*")

return 0;

else{

unsigned int num=0;

int j=31;

for(int i=0;i<Ip.length()-1;i++){

if(Ip.substr(i,1)=="1")

num+=pow(2,j);

j--;

}

return num;

}

}

unsigned int HextoInt(string protocol) {

int a,b;

if(protocol=="0x00/0x00")

return 0;

else {

if(protocol.substr(2,1)=="f")

a=15;

else if(protocol.substr(2,1)=="e")

a=14;

else if(protocol.substr(2,1)=="d")

a=13;

else if(protocol.substr(2,1)=="c")

a=12;

else if(protocol.substr(2,1)=="b")

a=11;

else if(protocol.substr(2,1)=="a")

a=10;

else{

a=atoi(protocol.substr(2,1).c_str());

}

if(protocol.substr(3,1)=="f")

b=15;

else if(protocol.substr(3,1)=="e")

b=14;

else if(protocol.substr(3,1)=="d")

b=13;

else if(protocol.substr(3,1)=="c")

b=12;

else if(protocol.substr(3,1)=="b")

b=11;

else if(protocol.substr(3,1)=="a")

b=10;

else{

b=atoi(protocol.substr(3,1).c_str());

}

return ((a*16)+(b*1));

}

}

void CPU(Tree * tree_1,Tree * tree_2, Matrix2 out,Matrix headers,Matrix rules,Matrix3 bitvs,Matrix3 bitvd,int numrule,Matrix3 abitvs,Matrix3 abitvd)

{

for(int index=0;index<headers.height;index++) {

int acm=0;

Tree *temp;//for rule

Tree *tree1;//for rule

Tree *tree2;//for rule

tree1=tree_1;

tree2=tree_2;

char src[32];

char dest[32];

int i=0;

for (int j = 32-1; j >= 0; --j) {

if (headers.elements[index] & (1 << j))

src[i]='1';

else{

src[i]='0';

}

i++;

}

i=0;

for (int j = 32-1; j >= 0; --j) {

if (headers.elements[index+headers.height] & (1 << j))

dest[i]='1';

else{

dest[i]='0';

}

i++;

}

//---------------------------------------------------------input Source

bitset<1000> s;

bitset<100> as;

int Ns,Nd;

int j=0;

while(j < 32 && tree1 != NULL){

if(tree1->rule != -1)//this node has a bitset

temp=tree1;

if(src[j] == '0' ){

tree1=tree1->left;

acm+=1;

} else if(src[j] == '1' ){

tree1=tree1->right;

acm+=1;

}

j++;

}

Ns = temp->rule;

//////////////////////////////////////////////////dst

j=0;

while(j < 32 && tree2 != NULL){

if(tree2->rule != -1)//this node has a bitset

temp=tree2;

if(dest[j] == '0' ){

tree2=tree2->left;

acm+=1;

}

else if(dest[j] == '1' ){

tree2=tree2->right;

acm+=1;

}

j++;

}

Nd=temp->rule;

for(int i=0;i<rules.height;i++){

if(bitvs.elements[Ns+bitvs.height*i]==1 && bitvd.elements[Nd+bitvd.height*i]==1)

s[i]=1;

else{

s[i]=0;

}

}

for(int i=0;i<abitvs.width;i++){

if(abitvs.elements[Ns+abitvs.height*i]==1 && abitvd.elements[Nd+abitvd.height*i]==1)

as[i]=1;

else{

as[i]=0;

}

}

j=0;

int f=0;

while(j<abitvs.width && f==0){

acm+=1;

if(as[j]==1){

for(int i=j*(rules.height/abitvs.width);i<j*(rules.height/abitvs.width)+10;i++){

acm+=1;

if(s[i]==1){

int nrule=i;

if((headers.elements[index+headers.height*4] == rules.elements[nrule+rules.height*4] || rules.elements[nrule+rules.height*4] == 0)// potocol

&& headers.elements[index+headers.height*2] >= rules.elements[nrule+rules.height*0] //&& sport >= fsport

&& headers.elements[index+headers.height*2] <= rules.elements[nrule+rules.height*1] //&& sport <= esport

&& headers.elements[index+headers.height*3] >= rules.elements[nrule+rules.height*2] //&& dport >= fdport

&& headers.elements[index+headers.height*3] <= rules.elements[nrule+rules.height*3]){ //&& dport <= edport

out.elements[index] = nrule;

f=1;

}

}

}

}//if as

j++;

}//while

out.elements[index+out.height]=acm;

}//for(all headers)

}

int main(void)

{

const int numrule = 1000;

const int nAggregate =10;

///////////////////////////////////////////////////////////open file

ifstream readf("ACL-ABVFilters1k",ios::in);

if(!readf){

cerr<<"File can not open!"<<endl;

exit(1);

}

string src,dst,fsport,esport,fdport,edport,mask,z,pro;

Matrix rules;

rules.width=5;

rules.height=numrule;

size_t size = rules.width * rules.height * sizeof(unsigned int);

rules.elements = (unsigned int *)malloc(size);

Matrix3 bitvs;

bitvs.width=numrule;

bitvs.height=numrule;

size = bitvs.width * bitvs.height * sizeof(bool);

bitvs.elements = (bool *)malloc(size);

Matrix3 bitvd;

bitvd.width=numrule;

bitvd.height=numrule;

size = bitvd.width * bitvd.height * sizeof(bool);

bitvd.elements = (bool *)malloc(size);

for(int i=0;i<numrule*numrule;i++){

bitvs.elements[i]=0;

bitvd.elements[i]=0;

}

Tree *tree1 = newTreeNode(-1);

Tree *found;

Tree *tree2 = newTreeNode(-1);

int js=0;

int jd=0;

clock_t start2;

clock_t end2;

for(int i=0;i<numrule;i++){

readf>>src>>dst>>fsport>>z>>esport>>fdport>>z>>edport>>pro>>mask;

src=src.substr(1,src.length());

rules.elements[i+rules.height*0]=atoi(fsport.c_str());//first sport

rules.elements[i+rules.height*1]=atoi(esport.c_str());//end sport

rules.elements[i+rules.height*2]=atoi(fdport.c_str());//first dport

rules.elements[i+rules.height*3]=atoi(edport.c_str());//end destination port

rules.elements[i+rules.height*4]=HextoInt(pro);//pro

bitset<numrule> b1;

////create tree

if(IPtoBin(src).length()-1==0){

found = tree1;

}else{

string Ips = IPtoBin(src).c_str();//ip source

found=insertTreeNode(tree1,atoi(Ips.substr(0,1).c_str()));//bit 1

if(tree1->rule != -1){

for(int t=0;t<numrule;t++){

if(bitvs.elements[tree1->rule+bitvs.height*t]==1)

b1[t]=1;

}

}

int j=1;

while(Ips.substr(j,1) != "*"){ //rest bits

if(found->rule != -1){

for(int t=0;t<numrule;t++){

if(bitvs.elements[found->rule+bitvs.height*t]==1)

b1[t]=1;

}

}

found=insertTreeNode(found,atoi(Ips.substr(j,1).c_str()));

j++;

}

}

if(found->rule==-1){

found->rule=js;

js++;

}

bitvs.elements[found->rule + bitvs.height*i]=1;

for(int t=0;t<numrule;t++){

if(b1[t]==1)

bitvs.elements[found->rule+bitvs.height*t]=1;

}

stack<Tree*> s;

stack<Tree*> s1;

s.push(found); s1.push(found);

while(!s1.empty()) {

Tree *temp = s1.top(); s1.pop();

if(temp->left!= NULL) {

s.push(temp->left);

s1.push(temp->left);

}

if(temp->right != NULL) {

s.push(temp->right);

s1.push(temp->right);

}

}

while(!s.empty()) {// s is list of all child nodes

Tree *temp = s.top(); s.pop();

if(temp->rule != -1){

for(int t=0;t<numrule;t++){

if(bitvs.elements[found->rule + bitvs.height*t] == 1)

bitvs.elements[temp->rule+bitvs.height*t]=1;

}

}

}

///////////////////////////////////////////////////////////////////////destination

bitset<numrule> b2;

if(IPtoBin(dst).length()-1==0){

found = tree2;

}

else{

string Ipd = IPtoBin(dst).c_str();//ip destination

found=insertTreeNode(tree2,atoi(Ipd.substr(0,1).c_str()));

if(tree2->rule != -1){

for(int t=0;t<numrule;t++){

if(bitvd.elements[tree2->rule+bitvd.height*t]==1)

b2[t]=1;

}

}

int j=1;

while(Ipd.substr(j,1) != "*"){

if(found->rule != -1){

for(int t=0;t<numrule;t++){

if(bitvd.elements[found->rule+bitvd.height*t]==1)

b2[t]=1;

}

}

found=insertTreeNode(found,atoi(Ipd.substr(j,1).c_str()));

j++;

}

}

if(found->rule==-1){

found->rule=jd;

jd++;

}

bitvd.elements[found->rule + bitvd.height*i]=1;

for(int t=0;t<numrule;t++){

if(b2[t]==1)

bitvd.elements[found->rule+bitvd.height*t]=1;

}

s.push(found); s1.push(found);

while(!s1.empty()) {

Tree *temp = s1.top(); s1.pop();

if(temp->left!= NULL) {

s.push(temp->left);

s1.push(temp->left);

}

if(temp->right != NULL) {

s.push(temp->right);

s1.push(temp->right);

}

}

while(!s.empty()) {// s is list of all child nodes

Tree *temp = s.top(); s.pop();

if(temp->rule != -1){

for(int t=0;t<numrule;t++){

if(bitvd.elements[found->rule + bitvd.height*t] == 1)

bitvd.elements[temp->rule+bitvd.height*t]=1;

}

}

}

}//all rules

///////////////////////////////////////////////////////////////// create Agregate BV

Matrix3 abitvs;

abitvs.width=(numrule/nAggregate);//****************

abitvs.height=numrule;

size = abitvs.width * abitvs.height * sizeof(bool);

abitvs.elements = (bool *)malloc(size);

Matrix3 abitvd;

abitvd.width=(numrule/nAggregate);//*******************

abitvd.height=numrule;

size = abitvd.width * abitvd.height * sizeof(bool);

abitvd.elements = (bool *)malloc(size);

for(int i=0;i<abitvd.width*abitvd.height;i++){

abitvs.elements[i]=0;

abitvd.elements[i]=0;

}

for(int i=0;i<numrule;i++){

for(int j=0;j<numrule/nAggregate;j++){

int f=0;

for(int k=0;k<nAggregate;k++){

if(bitvs.elements[i+bitvs.height*(k+(j*nAggregate))] == 1)

f=1;

}

if(f==1)

abitvs.elements[i+abitvs.height*j]=1;

}

}

for(int i=0;i<numrule;i++){

for(int j=0;j<numrule/nAggregate;j++){

int f=0;

for(int k=0;k<nAggregate;k++){

if(bitvd.elements[i+bitvd.height*(k+(j*nAggregate))] == 1)

f=1;

}

if(f==1)

abitvd.elements[i+abitvd.height*j]=1;

}

}

//////////////////////////////////////////////////////////////////////////

cout<<"size of tree1: "<<treeSize(tree1)<<endl;

cout<<"size of tree2: "<<treeSize(tree2)<<endl;

int const n1=8545;

int const n2=8004;

Tree * tree_d1;

Tree * tree_d2;

cudaMalloc((void **)&tree_d1, n1 * sizeof(Tree));

cudaMalloc((void **)&tree_d2, n2 * sizeof(Tree));

Tree tree1_1[n1];

Tree * p1 = tree_d1;

traverse(tree1,tree1_1,p1);

Tree tree2_2[n2];

Tree * p2 = tree_d2;

traverse(tree2,tree2_2,p2);

/////////////////////////////////////////////////////////////////////////////

const int numtrace=1000;

Matrix headers;

headers.width=5;

headers.height=numtrace;

size = headers.width * headers.height * sizeof(unsigned int);

headers.elements = (unsigned int *)malloc(size);

ifstream readtrace("ACL-ABVFilters1k_trace1k",ios::in);

unsigned int s,d,sp,dp,pr,z1;

for(int i=0;i<numtrace;i++)

{

readtrace>>s>>d>>sp>>dp>>pr>>z1>>z1;

headers.elements[i]=s;

headers.elements[i+numtrace]=d;

headers.elements[i+numtrace*2]=sp;

headers.elements[i+numtrace*3]=dp;

headers.elements[i+numtrace*4]=pr;

}

int * output_d;

cudaMalloc((void **)&output_d, numtrace* sizeof(int));

Matrix2 output;//answer rules

output.width=2;

output.height=numtrace;

size = output.width * output.height * sizeof(int);

output.elements = (int *)malloc(size);

Matrix d_r;

d_r.width = rules.width; d_r.height = rules.height;

size = d_r.width * d_r.height * sizeof(unsigned int);

cudaMalloc((void **)&d_r.elements, size);

Matrix3 d_bvs;

d_bvs.width = bitvs.width; d_bvs.height = bitvs.height;

size = d_bvs.width * d_bvs.height * sizeof(bool);

cudaMalloc((void **)&d_bvs.elements, size);

Matrix3 d_bvd;

d_bvd.width = bitvd.width; d_bvd.height = bitvd.height;

size = d_bvd.width * d_bvd.height * sizeof(bool);

cudaMalloc((void **)&d_bvd.elements, size);

Matrix3 d_abvs;

d_abvs.width = abitvs.width; d_abvs.height = abitvs.height;

size = d_abvs.width * d_abvs.height * sizeof(bool);

cudaMalloc((void **)&d_abvs.elements, size);

Matrix3 d_abvd;

d_abvd.width = abitvd.width; d_abvd.height = abitvd.height;

size = d_abvd.width * d_abvd.height * sizeof(bool);

cudaMalloc((void **)&d_abvd.elements, size);

for(int i=0;i<numtrace*2;i++)

output.elements[i]=-1;

Matrix d_h;

d_h.width = headers.width; d_h.height = headers.height;

size = headers.width * headers.height * sizeof(unsigned int);

cudaMalloc((void **)&d_h.elements, size);

//////////////////////////////////////////////////////////////////////////

dim3 dimBlock(768);

dim3 dimGrid(4);

float g=0;

float k=0;

float t=0;

float c=0;

cudaEvent_t start1,stop1;

cudaEvent_t start,stop;

cudaEventCreate(&start1);

cudaEventCreate(&stop1);

cudaEventCreate(&start);

cudaEventCreate(&stop);

cudaEventRecord(start,0);

cudaMemcpy(d_h.elements, headers.elements, headers.width * headers.height * sizeof(unsigned int),cudaMemcpyHostToDevice);

cudaMemcpy(d_r.elements, rules.elements, d_r.width * d_r.height * sizeof(unsigned int) ,cudaMemcpyHostToDevice);

cudaMemcpy(d_bvs.elements, bitvs.elements, d_bvs.width * d_bvs.height * sizeof(bool) ,cudaMemcpyHostToDevice);

cudaMemcpy(d_bvd.elements, bitvd.elements, d_bvd.width * d_bvd.height * sizeof(bool) ,cudaMemcpyHostToDevice);

cudaMemcpy(d_abvs.elements, abitvs.elements, d_abvs.width * d_abvs.height * sizeof(bool) ,cudaMemcpyHostToDevice);

cudaMemcpy(d_abvd.elements, abitvd.elements, d_abvd.width * d_abvd.height * sizeof(bool) ,cudaMemcpyHostToDevice);

cudaMemcpy(tree_d1, tree1_1, n1 * sizeof(Tree), cudaMemcpyHostToDevice);

cudaMemcpy(tree_d2, tree2_2, n2 * sizeof(Tree), cudaMemcpyHostToDevice);

cudaMemcpy(output_d, output.elements, numtrace*sizeof(int),cudaMemcpyHostToDevice);

cudaEventRecord(start1,0);

kernel<<<dimGrid, dimBlock>>>(tree_d1,tree_d2, output_d, d_h,d_r,d_bvs,d_bvd,d_abvs,d_abvd);

cudaEventRecord(stop1,0);

cudaEventSynchronize(stop1);

cudaMemcpy(output.elements, output_d, numtrace* sizeof(int), cudaMemcpyDeviceToHost);

cudaEventRecord(stop,0);

cudaEventSynchronize(stop);

float elapsedTime1;

cudaEventElapsedTime(&elapsedTime1,start1,stop1);

k+=elapsedTime1;

float elapsedTime;

cudaEventElapsedTime(&elapsedTime,start,stop);

g+=elapsedTime;

t+=elapsedTime-elapsedTime1;

cudaFree(d_h.elements);

cudaFree(d_r.elements);

cudaFree(d_bvs.elements);

cudaFree(d_bvd.elements);

cudaFree(d_abvs.elements);

cudaFree(d_abvd.elements);

cudaFree(output_d);

//////// CPU

for(int i=0;i<numtrace*2;i++)

output.elements[i]=-1;

start2 = clock();

CPU(tree1,tree2,output,headers,rules,bitvs,bitvd,numrule,abitvs,abitvd);

end2 = clock();

c+=(float)(end2 - start2) / (float)(CLOCKS_PER_SEC / 1000.0);

int Maxacm,Minacm,Avgacm;

Maxacm=Minacm=Avgacm=output.elements[numtrace];

for(int i=numtrace+1;i<numtrace*2;i++){

if(output.elements[i]<Minacm)

Minacm=output.elements[i];

if(output.elements[i]>Maxacm)

Maxacm=output.elements[i];

Avgacm+=output.elements[i];

}

cout <<"ACL1k-Result-Rules:"<<numrule<<" ,nAggregate: "<<nAggregate <<"- Number of Packets: " <<numtrace<<"\n";

cout<<"----------Access Memory----------"<<endl;

cout<<"Bit-Vector Max Access Memory: "<<Maxacm<<endl;

cout<<"Bit-Vector Min Access Memory: "<<Minacm<<endl;

cout<<"Bit-Vector Avg Access Memory: "<<Avgacm/numtrace<<endl;

cout<<"Bit-Vector All Access Memory: "<<Avgacm<<endl;

cout<<"-------------------------------------------------------"<<endl;

cout<<"----------Memory Space----------"<<endl;

cout<<"Memory useage(KB): "<<(numtrace*20 + numrule*20 + (n1+n2)*16 + numtrace*4 + 2*(bitvd.width*bitvd.height)+2*(abitvd.width*abitvd.height))/1024<<endl;//headers+rules+tree+result array+ bitvs&d+ abitv

cout<<"-------------------------------------------------------"<<endl;

cout<<"----------CPU----------"<<endl;

cout<<"Time CPU(ms): "<<c<<endl;

cout<<"throughput cpu(packet per second): "<<numtrace*1000/c<<endl;

cout<<"-------------------------------------------------------"<<endl;

cout<<"----------GPU----------"<<endl;

cout<<"Time kernel(ms): "<<k<<endl;

cout<<"Time Transfer(ms): "<<t <<endl;

cout<<"-------------------------------------------------------"<<endl;

cout<<"speedup: "<<c/k <<endl;

cout<<"throughput gpu(packet per second): "<<numtrace*1000/k<<endl;

getchar();

return 0;

}
